# Parents’ Perspectives on the Benefits of Animal-Assisted Intervention: A Systematic Review

**DOI:** 10.3390/bs15121663

**Published:** 2025-12-02

**Authors:** Francisco González-Sala, Karel Llopiz-Guerra, Ainhoa Ferri, Manuel Martí-Vilar

**Affiliations:** 1 Department of Developmental and Educational Psychology, Universitat de València, 46010 Valencia, Spain; francisco.gonzalea-sala@uv.es; 2 Department of Special Education, Universidad Central “Marta Abreu” de las Villas, Carretera de Camajuaní Km 5 1/2, Santa Clara 50100, Villa Clara, Cuba; karelllopizguerra@gmail.com; 3 Fundació Mira’M, Carrer de Rugat, 4-6, Camins al Grau, 46021 Valencia, Spain; ainhoa.ferri@fundaciomiram.org; 4 Department of Basic Psychology, Universitat de València, 46010 Valencia, Spain

**Keywords:** animal-assisted intervention, neurodevelopmental disorders, parents, systematic review

## Abstract

Animal-assisted intervention is widely used in children and adolescents with neurodevelopmental disorders. The aim of this review is to understand the perceptions of parents of children with neurodevelopmental disorders, regarding the benefits of their children’s participation in animal-assisted intervention. Using the PRISMA methodology, a search was conducted in the Web of Science and ProQuest Central databases. The number of articles included in the review was 23 after applying the selection criteria. The results indicate that in all interventions carried out with children with neurodevelopmental disorders, parents perceive improvements in their children mainly at the physical, social and emotional levels. In addition, they identify positive aspects that influence family functioning. It can be concluded that these types of interventions, regardless of the type of animal or activity, are a beneficial tool when addressing different symptoms associated with neurodevelopmental disorders, having an impact not only on the child or adolescent, but also on parents or caregivers.

## 1. Introduction

Neurodevelopmental disorders are characterised by the presence of a delay or impairment that hinders or prevents the acquisition of a whole range of skills related to different areas of development (Thapar et al., 2017), encompassing a wide variety of disorders, including intellectual disability, Attention Deficit Hyperactivity Disorder, Communication Disorders, Motor Disorders, Specific Learning Disorders and Autism Spectrum Disorder (APA, 2013), among others.

The IAHAIO (2018) defines Animal-Assisted Interventions (AAIs) as organised interventions that intentionally incorporate animals in various settings, such as health, education and public services. These interventions pursue specific objectives with the aim of improving people’s quality of life and achieving therapeutic benefits.

AAI is known in the health field as Animal-Assisted Therapy (AAT), a term coined by Levinson (1969). This is considered the starting point in the development of AAI programmes. Likewise, in 1977, several psychiatrists, veterinarians, and doctors created the Delta Foundation in Portland, United States. This organisation served to create the Delta Society (now Pet Partners) in 1981, considered the global pioneer in the development of research and working protocols for AAI (Martos-Montes et al., 2015). Taking the Delta Society’s definitions (Fredrickson, 1992) as a reference, AAI is divided into different modalities according to the purpose of the intervention and the type of professionals involved: Animal-Assisted Activities (AAAs) are interventions carried out by a human–animal team in contexts covered by non-formal education. These interventions pursue recreational, motivational and educational goals, emphasising the spontaneous interactions that may occur between the animal and the participants in the activity, without having any specific objectives (IAHAIO, 2018). Animal-Assisted Education (AAE) as a pedagogical resource and Animal-Assisted Therapy (AAT) have a clearly therapeutic objective directed towards the person. In line with these views, Lasa et al. (2011) classify three types of actions in relation to animal-assisted interventions (AAIs), namely service animal programmes, animal-assisted activities and animal-assisted therapies. According to Delgado et al. (2017), AAI focuses mainly on four areas or domains of development: psychomotor skills, cognitive skills, socio-emotional skills and language and communication skills, with the latter two areas being related to social development and sociability.

The Fundación Affinity (2023) details the various benefits that people who participate in AAI obtain. These include an increased desire and willingness to engage in group activities and teamwork, increased self-esteem (Arkow, 2010; Parra, 2016), as well as increased sense of responsibility (Fundación Affinity, 2009; Jacobsen, 1997). It also helps reduce anxiety and/or feelings of loneliness (Levinson, 1995; Signes & Rodrigo, 2010), opens up space for the expression of affection and feelings (Poresky, 1996; Fundación Affinity, 2009), increases levels of attention and concentration (Beetz, 2013; Parra, 2016), helps to overcome fears, improves physical condition, moderates anxiety levels, provides stress relief and physical benefits such as reduced heart rate and reduced blood pressure (Morrison, 2007). Focusing on neurodevelopmental disorders, there are various studies that show benefits in children after their participation in an AAI, such as Ávila-Álvarez et al. (2022), Chadwick et al. (2022) or Narvekar and Krishnan (2024). In the specific case of Hippotherapy, a form of horse-assisted therapy that focuses on neurological functioning and sensory processing (Koca & Ataseven, 2015), studies such as those conducted by Ajzenman et al. (2013), Muñoz-Lasa et al. (2011) and Snider et al. (2007) have found positive effects in children with ASD and cerebral palsy, while Silkwood-Sherer et al. (2012) obtained positive results with the implementation of this kind of therapy in children with Down syndrome.

Therapies that use dogs, both assistance dogs, which are trained for the purpose of specialised intervention with a specific group of individuals, and companion dogs, which are trained by the parents themselves or by private trainers, also report positive psychosocial outcomes in children with autism (Fung, 2017; Grandgeorge et al., 2012; Hardy & Weston, 2020; Guay et al., 2022; Hellings et al., 2022; Tseng, 2023), among others. These benefits are reflected in the regulation of emotional state and increase in social skills, along with the reduction in stress and anxiety levels, which can be high in children with ASD, contributing to calm children in the presence of and in contact with the dog (Appleby et al., 2022; Burgoyne et al., 2014; Carlisle et al., 2018; Hallyburton & Hinton, 2017; Narvekar & Narvekar, 2022; Viau et al., 2010).

The benefits of AAI also extend to parents or primary caregivers, as pointed out by Bibbo et al. (2019), Brown (2017) and Fecteau et al. (2017), reducing stress levels in parents and improving life quality.

The overall objective of this review is to conduct a systematic review of the scientific literature in order to understand the perceptions of parents of children with neurodevelopmental disorders, regarding the impact of AAI on their children and on family functioning.

Based on this general objective, the following specific objectives and corresponding hypotheses are proposed:

- To understand parents’ perceptions of the impact of AAI on their children in relation to social, emotional and motor skills, among others. In this regard, it is expected that parents identify benefits associated with AAI, mainly in psychosocial development (Hypothesis 1).
- To understand the effects that AAI may have on variables related to family functioning in general and on parents in particular, such as stress and anxiety, satisfaction or well-being. In this regard, it is expected that AAI will have positive effects on different aspects of family functioning according to parents’ perceptions (Hypothesis 2).

## 2. Materials and Methods

### 2.1. Search Strategy

For the correct preparation of the systematic review, a research question was formulated in PICO format, which was as follows: What impact does AAI have on parents of children and adolescents with neurodevelopmental disorders who participate in this type of intervention, and what are their perspectives on the therapy? The guidelines proposed in the PRISMA 2020 Statement (Page et al., 2021) were followed while preparing this review. The analysis process to determine whether the articles were appropriate for the research objective was carried out independently by two researchers acting as ‘blind’ evaluators. The COVIDENCE programme was used for this purpose, which increased inter-rater reliability, obtaining a reliability of 0.91. In cases of disagreement, a third reviewer was responsible for evaluating the article. After this process, a discussion was held to decide whether to include the study in the review or not.

This study includes a systematic review of the scientific literature related to AAI aimed at children with neurodevelopmental disorders. The review protocol was registered in PROSPERO with the following registration number: CRD42023446582.

The search was conducted in the Web of Science Core Collection and ProQuest databases in July 2025, including all works published up to 2024. The search equation used was in the Topic field: animal-assisted intervention* OR animal-assisted therapy OR animal-assisted education OR animal-assisted activities AND neurodevelopmental disorder* OR ASD OR Autism OR Asperger OR ADHD OR Attention deficit disorder and hyperactivity OR learning disorders OR Down Syndrome.

### 2.2. Selection Criterion

The following inclusion criteria were selected: quantitative and qualitative empirical studies addressing parents’ perceptions of the effect of AAI; published and peer-reviewed; written in English or Spanish; focusing on children and adolescents with neurodevelopmental disorders; published up to July 2025. The exclusion criteria were as follows: review studies or meta-analyses; theoretical articles; other types of documents such as books, book chapters, conference proceedings or doctoral theses; studies that do not address parents’ perceptions of the effects of AAI in their results; those using samples of adults and/or children and adolescents with mental or physical health problems, acquired brain damage or traumatic experiences.

### 2.3. Procedure

**Figure 1:**
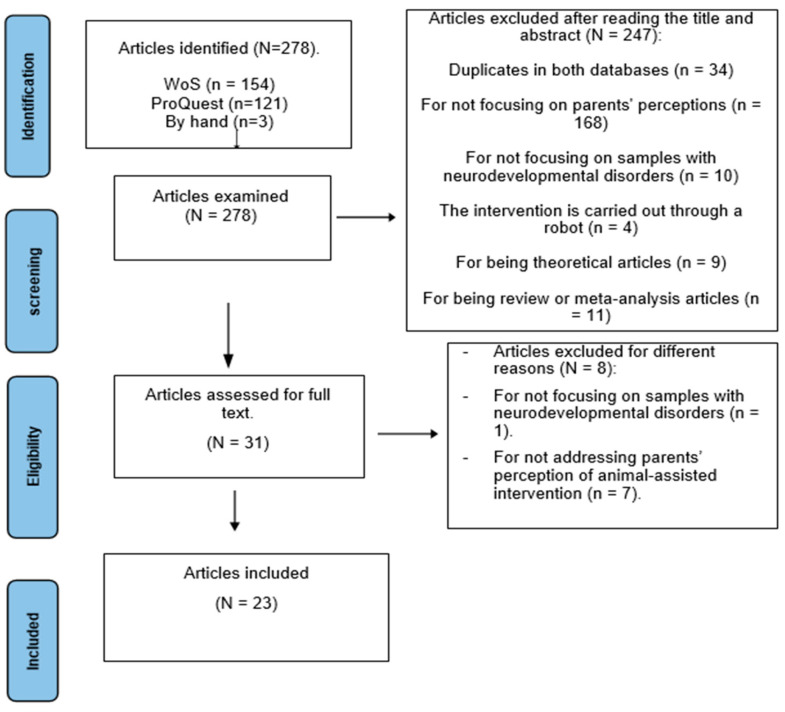
Flowchart of the different phases carried out in the review.

The total number of papers analysed was 278, of which 154 were found in WoS, 121 in ProQuest, and 3 articles were manually included when reviewing the references. After removing duplicate articles in both databases (n = 34), the titles and abstracts of a total of 244 articles were read, and a total of 213 studies were excluded for various reasons: not focusing on parents’ perceptions (n = 168), not focusing on samples with neurodevelopmental disorders (n = 10), the intervention was performed by a robot (n = 4), because they were theoretical articles (n = 9), because they were review articles or meta-analyses (n = 11), because they focused on therapists’ perceptions (n = 5), and because they focused on samples of people over 18 years old (n = 15). After this, a total of 31 articles were read in full, eliminating one study for not including children or adolescents with neurodevelopmental disorders and seven for not including parents’ perceptions in accordance with the object of study of this review. The number of articles included in this review was 23. This process is shown in Figure 1.

### 2.4. Methodological Quality of the Selected Articles

Two of the authors of this study reviewed the quality of the articles included in the review, based on the Standards for QUality Improvement Reporting Excellence 2.0 (QUIRE 2.0) scale by (Ogrinc et al., 2016). To this end, the 18 indicators included in the scale were selected, relating to the sections title and abstract, introduction, method, results, discussion and other information, classifying the articles into three categories: high quality (when between 12 and 18 indicators were met), medium quality (between 11 and 6 indicators) and low quality (5 or fewer indicators). After application, all articles were assessed as high quality, although the indicator referring to the description of the intervention was the one with the most shortcomings, as some articles did not fully describe the methodology used in the AAI programmes. The inter-rater agreement between the two reviewers, according to Cohen’s Kappa coefficient (Orwin, 2018), was 0.88, indicating very good agreement between the reviewers (Hernández-Nieto, 2002).

## 3. Results

Table 1 summarizes the main characteristics of the 23 studies included in this structured review in chronological and alphabetical order.

### 3.1. Characteristics of the Samples and Type of AAI

Considering the type of population targeted by the interventions, it should be noted that in the vast majority of cases, 20 studies (86.9%), these interventions were aimed at children with ASD, with three studies, Boyd and Le Roux (2017), Scotland-Coogan et al. (2021) and Stumpf and Breitenbach (2014), including children with other types of disorders and disabilities in addition to this type of disorder.

Regarding the origin of the samples, these are distributed across a total of nine countries. Specifically, eight studies were conducted in Australia, five in the United States, three in the United Kingdom, two in Ireland, one in Canada (Guay et al., 2024), one in South Africa (Boyd & Le Roux, 2017), one in the Netherlands (van der Steen et al., 2019), one in Germany (Stumpf & Breitenbach, 2014), and one in Brazil (Michelotto et al., 2019).

Depending on the type of AAI, 15 studies (65.2%) use dogs in two different ways: either through occupational therapy or assistance dogs or through pet dogs. The use of horses in therapy is present in six studies (20.1%), one of which specifies Hippotherapy as the type of intervention (Scotland-Coogan et al., 2021). Finally, one study uses dolphins (Stumpf & Breitenbach, 2014), and another uses farm animals (Dolecheck et al., 2024).

### 3.2. Benefits of AAI in Children with Neurodevelopmental Disorders, as Perceived by Their Parents or Caregivers

Depending on the type of therapy, the studies included in this review point out benefits to children in different areas of development, according to their parents.

In relation to the use of therapy involving horses, parents perceive benefits in the physical and motor domain, with improvements in body posture (Boyd & Le Roux, 2017), stability and balance (Boyd & Le Roux, 2017; Scotland-Coogan et al., 2021; Cleary et al., 2024), increased strength and fine/gross motor skills (Scotland-Coogan et al., 2021). All these findings show an impact on greater autonomy in actions or tasks such as walking, dressing and showering.

In studies that use horses in therapy, parents perceive improvement at the social level: Boyd and Le Roux (2017), Malcolm et al. (2018), Tan and Simmonds (2018), van der Steen et al. (2019), Kalmbach et al. (2020), Scotland-Coogan et al. (2021) and Cleary et al. (2024). These benefits include greater confidence and interest in social relationships, with participants becoming more communicative, more aware of others and experiencing fewer relationship problems. These benefits are also linked with language improvement, in speech and receptive language (Boyd & Le Roux, 2017; Scotland-Coogan et al., 2021) and language acquisition (Scotland-Coogan et al., 2021). Emotionally, parents perceive their children to be in a better mood (Tan & Simmonds, 2018; Kalmbach et al., 2020), with greater feelings of joy and happiness (Boyd & Le Roux, 2017; Tan & Simmonds, 2018; Kalmbach et al., 2020; Cleary et al., 2024), greater emotional regulation, appearing calmer and more relaxed (Boyd & Le Roux, 2017; Malcolm et al., 2018; Tan & Simmonds, 2018; van der Steen et al., 2019; Kalmbach et al., 2020; Scotland-Coogan et al., 2021; Cleary et al., 2024), which lead parents to perceive a decrease in emotional problems (van der Steen et al., 2019) and improved self-esteem (Boyd & Le Roux, 2017; Tan & Simmonds, 2018).

**Table 1:** Main characteristics and results found through the systematic review.

Study	Aim	Country/Sample	Design/Instruments	AAT Program or Action	Parents’ Perceptions: Main Outcomes
Burgoyne et al. (2014)	Parents’ perspective on the impact of the assistance dog on children with ASD.	Ireland Experimental group: n = 134 parents/guardians with an assistance dog. Control group: n = 87 parents of children on the waiting list were surveyed.	Quantitative and qualitative design. A cross-sectional study. Perceived Competence Scales (PCS) (Williams et al., 1998). Caregiver Strain Questionnaire (CGSQ) (Brannan et al., 1997). In addition, questions about parents’ perception of safety and a section on benefits and limitations are included.	Assistance dog.	The experimental group reported greater perceptions of safety regarding environmental risks (M = 32.43; *p* < 0.001) compared to the control group (M = 22.97), especially in cases where children were enrolled in special education schools (Mdif. = 6.62; 95% CI 0.639 to 12.61), with no differences observed for children attending regular schools. At the social level, parents in the experimental group perceive that people respond more respectfully and responsibly to their children in public places (M = 15.87, *p* < 0.001) compared to the control group (M = 10.67). This perception is higher and significant among parents of children enrolled in special education schools (Mdif. = 6.65; 95% CI 3.79 to 9.51) and among those in mainstream schools with special education classrooms (Mdif. = 7.01; 95% CI 2.88 to 11.13) compared to children in regular mainstream schools. Parents in the experimental group perceive greater competence in managing their children (M = 19.75, *p* < 0.023) compared to the control group (M = 17.91). Qualitatively, parents from both groups identify safety-related benefits, especially when they are occupied with other tasks. Relationally, they highlight the friendship bond formed between the child and the dog and the child’s acquisition of responsibilities in caring for the dog. The experimental group notes increased social visibility and awareness of ASD, while the control group emphasizes greater sociability of the child through the dog both inside and outside the home and a reduction in the child’s anxiety. Concerns mostly relate to caring for the dog and the connection between the child and the dog if such a bond does not form.
Carlisle (2014)	Examine the decision-making process and parents’ perception of the benefits of having pet dogs in homes with children with autism.	United States Seventy parents (61 females, 8 males) of children with autism aged between 8 and 18 years participated. Among them, 47 belonged to the dog group and 23 to the non-dog group.	Cross-sectional descriptive design. Telephone interview. Sociodemographic data and open-ended questions about decision-making.	Pet Dog Ownership.	The reasons parents decide on pet dog ownership were “Always had a dog,” grew up with dogs (51.1%), parents like/love dogs (38.3%), teach children responsibility (31.9%), good for children (31.9%), children wanted a dog (27.6%), companion for children (27.6%), good for children with ASD (25.5%) and teach compassion/calm the child (19.1%). Parents in the dog group identified benefits such as teaching responsibility (51.1%), companionship (46.8%), calming/stress relief (29.8%), entertainment/happiness (34%), unconditional love (25.5%), promotion of social interaction (14.9%), teaching empathy/tolerance (14.9%) and protection/safety (14.9%). Regarding burdens, they noted the cost and time of care and travel limitations.
Stumpf and Breitenbach (2014)	Parents’ perspectives on the impact of Animal-Assisted Therapy (AAT) on their children and on the parents’ quality of life.	Germany N = 47 parents of children with disabilities between 5 and 10 years. Experimental group (n = 31) new dolphin-assisted therapy program—Down syndrome (n = 5), physical (n = 11) and mental retardation (n = 15), of which 54.8% are males. Control group (n = 16) non-treatment—Down syndrome (n = 4), physical (n = 5) and mental retardation (n = 7), of which 62.5% are males.	Pre-test/post-test design with control and experimental group. Interview families. Questionnaire about Child’s Social-Emotional Behaviour (Breitenbach et al., 2009); Questionnaire about Parental Quality of Life (Averbeek et al., 1997).	Dolphin-Assisted Therapy. Ten days. (First day diagnostic interviews; 4 days interaction with the dolphins in sessions lasting between 25 and 30 min. Sixth day recreation and group discussion. From day 7 to 10, parents are included in the sessions with the dolphins).	The experimental group reports improvements in communicative skills both short- and long-term in verbal communication (d = 0.76 and d = 0.81), non-verbal communication (d = 0.90), and self-initiated non-verbal communication (d = 0.54 and d = 0.61). When comparing groups, improvements remain significant short-term (*p* = 0.04, d = 0.84) and long-term (*p* = 0.03, d = 0.84) in the factor of non-verbal communication. For other factors, improvements are maintained short-term in verbal communication (*p* = 0.03, d = 0.82) and self-initiated non-verbal communication (*p* = 0.02, d = 0.52). Parents report improvements in social-emotional skills both short-term and long-term (*p* = 0.08, d = 0.63; *p* = 0.01; d = 0.99), self-confidence skills (*p* = 0.06, d = 0.60; *p* = 0.01, d = 0.58), and in sociability long-term (*p* = 0.04, d = 0.29). In parental quality of life, benefits are perceived in prevailing mood (F = 3.76, df = 34, *p* = 0.03) both short-term (d = 0.49) and long-term (d = 0.40), and in view of life (F = 3.42, df = 32, *p* = 0.05) short-term (d = 0.80), although this significant effect does not remain when using paired samples.
Hall et al. (2016)	Study the long-term effects on the family after acquiring a dog.	United Kingdom N = 37 parents of children with autism aged between 3 and 16 years. Experimental group: n = 22. Control group: n = 15.	Family functioning (Brief FAM-III-General Scale) (Skinner et al., 1995). Parenting Stress Index-Short Form (PSI-SF) (Abidin, 1995). Attachment to the dog: Lexington Attachment to Pets Scale (LAPS; Johnson et al., 1992).	Pet dog.	The experimental group shows a decrease in difficulties and an increase in family strengths, which leads to better family functioning compared to the control group (F (1, 32) = 4.71, *p* = 0.037, ηp^2^ = 0.125). The experimental group reports fewer family difficulties between time 1 and time 2 of the evaluation (t (21) = 2.69, *p* = 0.01). No significant differences are observed in the control group (t(13) = 0.34, *p* = 0.73). Although parental stress decreases in both groups, this reduction is greater in the experimental group, where 20% of parents move from a clinical level to a normal level of stress (χ^2^ (1, 34) = 3.17, *p* = 0.07). The largest differences occur in the total stress factor between the two evaluation points, with a difference of 10.9 points in the experimental group compared to 4.43 points in the control group. The experimental group also shows a decrease in dysfunctional interactions between parents and children.
Boyd and Le Roux (2017)	To know the perceptions of parents with children with disabilities about therapeutic horseback riding.	South Africa Twelve parents (11 females, 1 male) of children with disabilities aged between 6 and 12 years.	Exploratory qualitative research. Parent interviews (six questions) related to experiences in the program and its benefits and limitations.	Therapeutic horseback riding.	Positive psychosocial effects include increased confidence and independence as children feel they have control over the horse, feelings of pride and enhanced self-esteem, and feelings of joy and happiness. Cognitively, there is improved concentration, which allows better academic performance, and language improvements that boost social confidence and the ability to interact with others. Physically, there are improvements in posture, stability, muscle tone and rigidity, which facilitate walking in children. Additionally, parents report that their children are calmer and more relaxed, especially parents of children with ASD, ADHD and sensory issues. Some parents believe these benefits are directly related to their children’s participation in the intervention, while others cannot confirm this relationship.
Malcolm et al. (2018)	To understand the perceptions of professionals and parents of children with autism regarding the benefits and limitations of equine therapy.	United Kingdom Nine interviews were conducted with therapists, volunteers, teachers and parents of children with ASD.	Malcolm’s ‘observant participation’ (Moeran, 2009).	Horse-assisted therapy involved various games with the horse while therapists encouraged the child’s verbal communication toward the horse.	Parents reported improvements in social functioning, noticing their children becoming more communicative, increasing their comprehension, demonstrating better communication skills and becoming more aware of themselves and others.
Tan and Simmonds (2018)	Parents’ perceptions of the psychosocial impact of equine-assisted interventions on their children.	Australia Parents of six children (five girls and one boy) with ASD aged between 3 and 14 years old.	Qualitative research Semi-structured interview with related questions about the psychosocial benefits observed in their children and how they are aware of these benefits.	Equine-assisted interventions.	Parents perceive improvements in their children’s self-concept and emotional well-being, mainly in mood and self-regulation skills, with children appearing calmer and more relaxed and exhibiting improved behaviour. On a social level, parents perceive improvements in the quality of interactions and in their motivation to engage in them. They also point to the positive social climate of the intervention as having an effect on their well-being. They perceive benefits in discovering how their children enjoy themselves and appear happier, have less difficulty managing their behaviour, and generalise the skills learned to other contexts. It should be noted that some of these benefits are mainly perceived on the days when the children have sessions.
Harwood et al. (2019)	Explore the impact of companion canines on children with ASD.	Australia Eleven mothers of children with ASD aged between 5 and 12 years (7 males, 6 females).	Semi-structured interview about the child’s relationship with the dog and whether the dog influences the child emotionally and behaviourally.	Canine companion.	Ten of the eleven mothers describe the child’s relationship with the dog as positive. Nine mothers report that children show greater calm in stressful situations, which occurs through sensory experiences, such as touching the dog. In other cases, the dog is a distracting element that has a calming effect on the child. They report greater social connection through increased opportunities for communication related to the dog. They also associate the dog’s company with a positive effect on social skills, empathy and the ability to interpret non-verbal language. As negative aspects, they point out that in some cases children may behave inappropriately towards dogs.
Hill et al. (2020)	Knowing the parents’ perspective on animal-assisted occupational therapy intervention.	Australia Ten parents (nine mothers, one father) of children with ASD aged between 4 and 6 years and 11 months. Five parents formed the experimental group and five the control group.	Qualitative interpretive description design. Semi-structured interview.	Canine-assisted occupational therapy. Seven weekly sessions of 1 h duration (Hill et al., 2020).	The dog provides greater emotional security for the child, which facilitates the relationship between the child and the therapist. Parents indicate that the interaction with the dog helped their child to calm down and feel more comfortable during the session. These self-regulation skills generalize to other contexts. Additionally, they perceive an increased number of social behaviours directed towards the dog and the therapist, viewing this relationship with the dog as a friendship. All of this generates a greater sense of acceptance in the child and increased motivation to participate, even when activities were more challenging, paying more attention. They identify feelings of pride in their children when they perceive that goals set in the sessions have been achieved.
Michelotto et al. (2019)	Parents’ and therapists’ perception of the impact of animal-assisted activities (AAA) on their children’s behaviour.	Brazil Fifteen children with ASD. Fourteen boys and one girl. Aged 5.6 +/− 1.6 years.	The Autistic Behaviour Assessing Questionnaire (CACS-27) (Moraes, 1998).	AAA with dogs. Minimum of 10 weeks with 30 min sessions. Children interact freely with the dogs and other children in groups of 11.2 +/− 2.2 children per session.	Parents report a reduction in self-aggression and repetitive stereotyped movements, as well as positive effects on speech communication and creativity.
van der Steen et al. (2019)	Evaluate the effects of equine-assisted intervention in children with autism.	The Netherlands Mother (41 years old) and father (45 years old) of an 8-year-old girl with autism.	Semi-structured interview. Scale for Emotional Development—Revised (SED-R) (Vandevelde et al., 2015). Strengths and Difficulties Questionnaire (SDQ) Subscale (Parents) (Goodman, 1997). Video observations of the sessions.	Equine-assisted intervention. Five sessions, one per week, lasting between 90 and 120 min.	Regarding emotional development, the parents report significant positive changes between the pre-test and post-test in abilities associated with the following factors: Deal with her own body (Pre-test level 1; Post-test level 4), Emotion differentiation (Pre-test level 2; Post-test level 4) and Emotion regulation (Pre-test level 2; Post-test level 4). Concerning the SDQ, the mother reports a decrease in problems from pre-test to post-test in the factors (Total difficulties score 24–16; Emotional symptoms 8–4; Peer relationship problems 8–5), and an increase in Prosocial behaviour (5–8). For the father, the scores were (Total difficulties score 19–14; Peer relationship problems 8–4).
Kalmbach et al. (2020)	Describe the perceptions of parents of children with autism regarding occupational therapy in an equine environment.	United States Four mothers and one father of children with ASD aged between 8 and 13 years, all of whom are boys.	Qualitative descriptive research. Semi-structured interviews with the parents at 4–6 weeks and at 6 months after the intervention about their children’s experience and the impact on the child and family at the domestic, school, and community levels.	Occupational therapy in an equine environment. Sessions lasting between 45 and 60 min, once a week for 10 weeks.	Parental perspectives on the child’s experience: Importance of the horse and the therapist, such as physical contact with the horse, riding, children’s motivation, therapists’ knowledge of autism. Children’s positive emotions regarding their participation, such as excitement or enjoyment. Parental perspectives on the intervention in the daily lives of children and families: Positive impact on mood and social skills with parents and other family members. Reduction in hyperactivity or irritability and violent behaviour. All of this leads to greater satisfaction in the relationship with their children and greater self-control in parents.
London et al. (2020)	Parents’ perspectives on the impact of AAT on their children.	Australia Seventeen parents (3 males and 14 females) of children with ASD aged between 4 and 19 years, of whom 16 (94.1%) are boys.	Qualitative phenomenological study design. Semi-structured interview about decision-making, the child’s experience after the intervention and its effects.	AAT occupational therapy program with dogs. Five sessions, one per week for five weeks.	Mainly, parents decide to participate thinking about the achievements the child can reach with the intervention. Parents give positive evaluations regarding the fulfilment of objectives related to non-verbal communication and engagement and interaction (17, 100%), play and enjoyment (16, 94.1%), community participation (14, 82.4%), motivation (13, 76.5%), emotional regulation (12, 76.6%) and improving communication (12, 70.6%).
Scotland-Coogan et al. (2021)	To understand parents’ perceptions of the benefits of hippotherapy in children with disorders.	United States Eleven caregivers of 12 children with different disabilities (four females, eight males).	Qualitative multiple case study design. Semi-structured interviews (Stake, 1995).	Hippotherapy (equine-assisted treatment).	Caregivers report improvements in the following areas: Physical level—increased strength and flexibility, as well as enhanced skills related to fine and gross motor abilities, which make it easier for children to perform more tasks, such as dressing themselves. Improvements in balance and greater autonomy when walking. Language level—improvements in speech, receptive language, and increased communicative intentions. Psychology level—lower levels of frustration. Many parents note improvements associated with hippotherapy compared to other types of therapies they have experienced. Regarding quality of life, they highlight greater confidence and independence in their children when performing daily tasks on their own, which positively impacts the parents as they perceive their children are less dependent on them.
Ang and MacDougall (2022)	To know the perceptions of parents and therapists about the choice of AAT.	Parents of children with ASD (three males and one female) aged between 3 and 21 years and three therapists.	Qualitative phenomenological design. Semi-structured interviews with parents and therapists about their experiences and opinions of AAT.	Dogs.	Benefits of AAT according to parents. Physical: parents perceive the environment as safe and positive, which facilitates the child being more participative and receptive. Emotional: parents report a feeling of well-being as their child feels accepted, increased self-confidence, better impulse control (allowing the child to be less challenging), with benefits.
Appleby et al. (2022)	Parents of children with autism who own assistance dogs report numerous positive experiences.	Australia Eight mothers and fathers with children with autism aged between 7 and 12 years.	Qualitative descriptive design. Occupational mapping (Huot & Rudman, 2015). Semi-structured interviews about their experiences related to Autism Assistance Dogs.	Autism assistance dog.	Parents report that children gain freedom of movement, which in turn allows them to build strength for walking in a straight line and helps stretch the calf muscles. They feel safer when walking on the street because the children understand the dog’s signals related to traffic rules. Parents gain freedom to talk with others and feel more relaxed. Isolation is reduced as they can go to more places, spending more time outside the home. They perceive that others recognise the child’s behaviours as a result of their disorder, rather than a lack of discipline. The children appear calmer, with fewer and less severe behavioural crises related to stress. Additionally, the dog provides security and calmness to the child in stressful or difficult situations. Parents note that children form friendships with their dogs, sometimes even considering them confidants. They see benefits in language stimulation, the acquisition of social skills, and even an increase in empathy.
Richardson et al. (2022)	Parents’ perspectives on the impact of Animal Assisted Therapy (AAT) in their children are highlighted.	Australia Parents (n = 4) and mothers (n = 13) of children and adolescents with autism (3 girls and 13 boys) aged between 5 and 19 years.	Qualitative study. Semi-structured interview with 12 questions about parents’ perspectives on the Animal Assisted Therapy occupational therapy program.	AAT occupational therapy program (Groenewald, 2004) with dogs. Five sessions, one per week for five weeks. First session 90 min, four sessions 60 min.	Parents report positive effects in the following areas: stress management in children, participation and engagement, and social communication skills during the sessions. They also observe maintenance of self-care skills at 3, 6 and 9 months after the intervention.
Adkins et al. (2023)	To explore parents’ perceptions of how pet dogs in households can promote healthy nutrition and physical activity in children with ASD.	Australia Ten mother–child dyads of children with autism aged between 8 and 18 years.	Qualitative research. Semi-structured interviews conducted to understand the impact of pet dogs on the family and children with ASD.	Pet dogs.	Parents report feeling that their children are happier when playing with their dog, creating a bond between the child and the animal. They mention fewer effects related directly to autism symptoms, although some parents report increased verbal language use. Some negative aspects related to playing with the dog and behavioural issues associated with autism in interactions with the dog are mentioned to a lesser extent. They also note that the dog’s arrival helped introduce care routines in which the children participate and that they spend more time outside the home. Parents report increased awareness in their children about healthy nutrition and physical activity for both the child and the dog, although they note that the children do not enjoy physical activity much. Regarding nutrition, while children are more aware of healthy eating, they do not enjoy eating this type of food. However, the healthy diet has allowed the introduction of different foods and new textures.
Morgan and O’Byrne (2023)	To understand the experiences of parents of children with ASD, canine handlers, and teachers regarding assistance canines	Ireland Four parents of children with autism, three canine handlers, two teachers.	Qualitative research. Semi-structured interviews with parents about the relationship between the child and the dog and the benefits and limitations.	Assistance Canines.	Parents emphasize the importance of the bond formed between the child and the dog. They attribute positive aspects to this relationship, such as increased social interaction, greater confidence when doing things independently, companionship and improvements in language development, as well as a reduction in behavioural problems. At the family level, parents report an increase in activities outside the home and a reduction in stress levels related to caregiving.
Cleary et al. (2024)	To know the experiences of parents and the benefits after their children’s participation in horse-based therapies.	Australia Four mothers and two fathers of three boys and one girl with ASD. Eight staff members of equine therapy.	Qualitative research. Semi-structured interviews about the impact on their children related to their participation in horse riding.	Horse-based therapies.	Parents focus the impact of the therapy on the following: Physical and social benefits: an increase in the child’s sense of responsibility. Improvements in balance, communication skills, social skills and socialization; as a protective factor for mental health: learning to manage anxiety, increased resilience, better self-regulation, greater happiness and an increased sense of social support.
Dolecheck et al. (2024)	Parents’ perspectives on the benefits of farm-based Animal Assisted Therapy (AAT) for their children highlight several key points.	United States Five parents of children with autism.	Exploratory sequential design. Semi-structured interview.	Farm Animal-Assisted Therapy (cows, sheep, goats, horses and pigs).	Increases in social behaviours, regulation of behaviours and a sense of normalcy for the children.
Guay et al. (2024)	Parent perceptions about the acceptability and effects of having either an assistance dog or a companion dog for their children include several key insights.	Canada First phase: 85 parents (assistance group n = 57; companion dogs group n = 28) of children with autism aged between 3 and 17 years (M = 10.37, SD = 3.67), of which 98.8% were mothers. Second phase: N = 17 (assistance dogs n = 14, companion dogs n = 3).	Explanatory sequential design. French version of the Treatment Acceptability Rating Form—Revised (TARF-R) (Reimers et al., 1992). Semi-structured interview questionnaire about the benefits for the child and the family.	Assistance or companion dogs.	Parents in the assistance dog group report greater satisfaction with the treatment (M = 5.60, SD = 0.76) than the comparison group (M = 5.24, SD = 0.68), with these differences being significant (t(83) = −2.12, *p* = 0.037, d = 0.49). Satisfaction with the presence of a dog is higher among families with another child with a disorder, regardless of the group (t(83) = −2.26, *p* = 0.026, d = 0.49); within this, parents of children with ADHD are the least satisfied (t(83) = 2.46, *p* = 0.016, d = 0.53), with significant differences in the assistance dog group (t(55) = 2.16, *p* = 0.036, d = 0.57). Families with assistance dogs report lower satisfaction when there is comorbidity with a behavioural disorder (t(55) = 2.09, *p* = 0.041, d = 1.09). Both types of families (assistance and companion) perceive positive effects in their children related to better emotional regulation, specifically in managing anger and anxiety when doing activities with the dog, with a reduction in the frequency and intensity of emotional outbursts, and faster decreases in tantrums and aggression. These effects are also observed in other contexts. They report increased communication and social interactions mainly related to issues concerning the dog, which helps expand the child’s social network and reduce social isolation. Additionally, they note benefits in autonomy, routines and responsibilities acquisition. Other benefits extend to other family members, such as an increased number, duration and frequency of activities outside the home, which contributes to reduced stress among other family members. Families report happier moments of interaction with the dog, spending more time together, especially among siblings. There are benefits in the couple’s relationship, allowing them to sleep together again, which grants them higher quality time for themselves. The burdens associated with having the dog mainly relate to caregiving responsibilities.
Rodriguez et al. (2024)	Evaluate the effect of service dogs on children with autism and their parents.	United States A total of 75 families of children with autism (39 in the service dog group; 36 in the comparison group).	Cross-sectional design. Instruments: Child: SCQ; CSHQ; ABC; BASC-3; PRPP. Parents: CGSQ; PROMIS^®^; PHQ-9; PedsQL™; FIMFFS. Animal–human bond: MDORS (Monash Dog Owner Relationship Scale) and IOS (Inclusion of Other in the Self).	Service dogs	Regarding parent-related variables, there are no statistically significant differences between the experimental group and the control group in any of the analysed variables (Caregiver Strain Questionnaire, PROMIS Sleep Disturbance, Patient Health Questionnaire, PedsQL Family Impact—Daily Activities, and PedsQL Family Impact—Family Relationships). In terms of children’s scores, there are significant differences in the Children’s Sleep Habit Questionnaire (*p* = 0.038), Sleep Initiation and Duration (*p* = 0.005), and Sleep Anxiety/Co-Sleeping (*p* = 0.026), with lower mean scores in the experimental group. There is no relationship between having a dog and benefits in emotional self-control, withdrawal, irritability, or hyperactivity.

AAA (Animal-Assisted Activity). AAT (Animal-Assisted Therapy). D = effect size. SCQ (Social Communication Questionnaire). CSHQ (The Children’s Sleep Habit Questionnaire). ABC (Aberrant Behaviour Checklist). BASC-3 (The Behaviour Assessment Scale for Children 3^rd^ edition). PRPP (Peer Relationships Paediatric Parent-Proxy Short Form (7-A v2.0). CGSQ (The Caregiver Strain Questionnaire). PROMIS (The PROMIS^®^ Sleep Disturbance Short Form 6-A). PHQ-9 (The Patient Health Questionnaire-9). PedsQL™ (Paediatric Quality of Life Inventory). FIMFFS (Family Impact Module Family Functioning Scale). MDORS (The Monash Dog-Owner Relationship Scale). IOS (The Inclusion of Other in Self scale). TARF-R (Treatment Acceptability Rating Form—Revised).

In cases where dogs are part of the intervention, either in therapy or as companions, parents perceive benefits mainly in two areas: social and emotional. In the social sphere, they report greater sociability and social interaction (Burgoyne et al., 2014; London et al., 2020; Morgan & O’Byrne, 2023; Guay et al., 2024), which in turn can be linked to another aspect highlighted by parents, namely greater opportunities for communication and social participation (Harwood et al., 2019; Hill et al., 2020; London et al., 2020; Guay et al., 2024). The bond of friendship that develops with the dog has been noted in five of the studies (Burgoyne et al., 2014; Harwood et al., 2019; Appleby et al., 2022; Adkins et al., 2023; Morgan & O’Byrne, 2023), offering the child plenty of companionship (Carlisle, 2014), which helps combat the loneliness experienced by many children with ASD. Other aspects include an increase in social skills (Harwood et al., 2019) Appleby et al., 2022; Richardson et al., 2022) and empathy (Harwood et al., 2019; Appleby et al., 2022). On an emotional level, Burgoyne et al. (2014), Carlisle (2014), Harwood et al. (2019), Hill et al. (2020), London et al. (2020), Ang and MacDougall (2022), Appleby et al. (2022), Richardson et al. (2022) and Guay et al. (2024) report that parents perceive a decrease in anxiety in their children, which appears to be related to a decrease in behavioural problems (Appleby et al., 2022) and could be related to a decrease in self-harm and stereotypical movements typical of children with ASD (Michelotto et al., 2019). The increase in the sense of responsibility and commitment, mainly in caring for the dog, has also been noted by Burgoyne et al. (2014), Carlisle (2014), Richardson et al. (2022) and Guay et al. (2024), which allows children to establish more routines in their daily lives (Adkins et al., 2023; Guay et al., 2024). Interaction with dogs, according to parents’ perceptions, has also been associated with more positive emotional states, such as improved well-being, greater enjoyment of playing with the dog, increased emotional security and self-confidence, and a greater sense of pride (Hill et al., 2020; London et al., 2020; Ang & MacDougall, 2022). With regard to language development, the studies reviewed reported that parents perceived improvements in oral communication (Michelotto et al., 2019; Adkins et al., 2023) and in the interpretation of non-verbal language (Harwood et al., 2019; London et al., 2020). For Appleby et al. (2022) and Morgan and O’Byrne (2023), interaction with the dog stimulated the children’s language. Other benefits reported by parents included increased strength and greater freedom of movement (Appleby et al., 2022), improved sleep habits (Rodriguez et al., 2024), greater knowledge about healthy nutrition and the introduction of new foods into their children’s diets (Adkins et al., 2023), which occurs when children are involved in caring for the dog, including not only physical exercise but also the importance of healthy eating for the dog and for the child, with this involvement providing an opportunity for children to learn about healthy nutrition.

Other less frequent interventions that also reported positive perceptions by parents were those related to dolphin-assisted therapy (Stumpf & Breitenbach, 2014) and farm animal-assisted therapy (Dolecheck et al., 2024). Both types of intervention noted benefits in emotional regulation, social interaction, and verbal/non-verbal communication; the latter being mainly with dolphin therapy.

### 3.3. Benefits of AAI for Families of Children with Neurodevelopmental Disorders, as Perceived by Parents or Caregivers

At the family level, parents perceive a greater reduction in parental stress (Hall et al., 2016; Appleby et al., 2022; Morgan & O’Byrne, 2023; Guay et al., 2024). This can be linked to a reduction in problems in their relationship with their children (Hall et al., 2016; Guay et al., 2024), lessening of behavioural problems (Morgan & O’Byrne, 2023) and an increase in the sense of parental competence (Burgoyne et al., 2014) in families where the programme involved the participation of a dog. The presence of the dog also meant spending more time outdoors (Adkins et al., 2023; Morgan & O’Byrne, 2023; Guay et al., 2024). In addition, Guay et al. (2024) point out benefits in the couple’s relationship, as the child’s increased autonomy and the security provided by the dog allow them to spend more time together, while Appleby et al. (2022) highlight the value parents place on the freedom to talk to other people inside and outside home.

In the case of therapies that use horses, only two studies have reported benefits perceived by parents in terms of family functioning. Kalmbach et al. (2020) indicate greater parental satisfaction in terms of their relationship with their children and increased self-control, while Scotland-Coogan et al. (2021) refer to an increase in family life quality, related to greater autonomy and independence of their children when performing different tasks or activities.

## 4. Discussion and Conclusions

The aim of this review was to ascertain parents’ perceptions of the benefits in different areas of development in their children with neurodevelopmental disorders, after participating in some type of intervention or programme involving animals. All studies included in this review indicate that parents perceive benefits in different areas of development, mainly at the physical, social, language development and emotional levels, thus confirming the first hypothesis of this review. Specifically, one of the aspects most frequently mentioned by parents was improved emotional regulation, with parents reporting that their children appear calmer and more relaxed and showing less anxiety, regardless of the type of intervention or animal (Boyd & Le Roux, 2017; Malcolm et al., 2018; Tan & Simmonds, 2018; van der Steen et al., 2019; Kalmbach et al., 2020; Scotland-Coogan et al., 2021; Cleary et al., 2024; Burgoyne et al., 2014; Carlisle, 2014; Harwood et al., 2019; Hill et al., 2020; London et al., 2020; Ang & MacDougall, 2022; Appleby et al., 2022; Richardson et al., 2022; Guay et al., 2024; Dolecheck et al., 2024). In this regard, Solomon (2012) points out that in the case of children with ASD, interacting with an animal is easier because the person does not need to interpret what the animal is thinking, so the social communication difficulties experienced by children with ASD are diluted when they interact with animals, making interaction with animals easier than with humans (Grandin et al., 2015). This relationship, interpreted by parents as one of friendship and security, has been documented in the works of Burgoyne et al. (2014), Harwood et al. (2019), Appleby et al. (2022), Adkins et al. (2023) and Morgan and O’Byrne (2023), which may explain the large number of studies in this review where the intervention has been aimed at children with ASD.

Depending on the type of animal used, it should be noted that parents believe that interventions with horses have a greater physical and motor impact by increasing strength, improving posture and balance, if we take into account the number of studies that report these types of benefits (Boyd & Le Roux, 2017; Scotland-Coogan et al., 2021; Cleary et al., 2024), while in the case of dogs, only the study by Appleby et al. (2022) shows benefits in this regard. This is perfectly understandable when one considers that horse riding is a physical activity that requires a range of skills at this level.

These benefits are perceived not only in relation to the child or adolescent but also in relation to personal aspects of the parents and family functioning, regardless of the type of intervention (Burgoyne et al., 2014; Hall et al., 2016; Appleby et al., 2022; Adkins et al., 2023; Kalmbach et al., 2020; Scotland-Coogan et al., 2021; Morgan & O’Byrne, 2023; Guay et al., 2024), thus confirming the second of the hypotheses proposed.

These results are consistent with a large number of studies that point to psychosocial and health benefits for children and adolescents who have participated in activities or therapies with animals (Fundación Affinity, 2023; Ajzenman et al., 2013; Muñoz-Lasa et al., 2011; Snider et al., 2007; Silkwood-Sherer et al., 2012; Fung, 2017; Grandgeorge et al., 2012; Hardy & Weston, 2020; Guay et al., 2022; Hellings et al., 2022; Sissons et al., 2022; Rehn et al., 2023; Tseng, 2023; Appleby et al., 2022; Burgoyne et al., 2014; Carlisle et al., 2018; Hallyburton & Hinton, 2017; Narvekar & Narvekar, 2022; Viau et al., 2010), although a number of issues need to be taken into account in relation to the results obtained from parents’ perceptions.

In this regard, most studies employ a qualitative descriptive methodology, which, as Sandelowski (2000, 2009) points out, is an appropriate methodology for describing events, phenomena or experiences, such as the perceptions of parents of children who have directly or indirectly participated in an animal-related programme or therapy. In this regard, limitations related to qualitative studies using small sample sizes (Guay et al., 2024) should be taken into account, as well as the use of self-report measures, in which responses may be influenced by parents’ expectations and memory biases (Burgoyne et al., 2014).

It should also be noted that few of the studies included in this review used any type of objective to measure the benefits of the intervention; specifically, only eight studies did (Burgoyne et al., 2014; Stumpf & Breitenbach, 2014; Hall et al., 2016; Michelotto et al., 2019; van der Steen et al., 2019; Appleby et al., 2022; Guay et al., 2024; Rodriguez et al., 2024), which may be consistent with or differ from parents’ perceptions. Sample size may be a limitation, as pointed out by various studies (Cleary et al., 2024); although, as Braun and Clarke (2021) indicate, this does not necessarily mean that the results are not rigorous, as sample size may be determined by the relevance of the topic to the research question itself, meaning that small samples may also be perfectly valid. In this regard, various studies point to small sample sizes as one of the limitations that explain why some effects are not maintained in matched samples, as is the case in the study by Stumpf and Breitenbach (2014) on socio-emotional skills in children in the short term or on mood and outlook on life in parents six months after the intervention. Rodriguez et al. (2024) also attribute to sample size and sample heterogeneity, by not taking into account the different phenotypes of autism, the failure to find significant differences between matched samples in some of the variables assessed in children, such as withdrawal, negative emotionality, emotional self-control, hyperactivity, irritability and lethargy. In the case of parents’ perceptions of caregiver strain, there were no differences between the control group and the experimental group, which may be due to the increased level of stress associated with caring for the dog. Other factors in which there were no differences between groups were sleep disturbance, depression or family daily activities according to the severity of autism, which is attributed to aspects related to the type of instruments used to measure parents’ perceptions, as well as the high rates of sleep disorders in the matched samples or the bond established between the caregiver and the dog (Rodriguez et al., 2024).

Regarding the studies included in this review, in the case of the study by Stumpf and Breitenbach (2014), which includes dolphins in the IAA, in addition to the limitations the authors may point out in their study, it is important to consider a whole series of unethical aspects associated with captive breeding and the exploitation of dolphins, as well as the ineffectiveness of this type of intervention, as noted by Whale and Dolphin Conservation (2021). Specifically, in the Stumpf and Breitenbach (2014) study, the authors themselves indicate that the study was conducted following the review and evaluation of dolphin living conditions carried out by the European Association for Zoos and Aquariums. Considering other ethical aspects when animals are used in this type of intervention, it is worth highlighting the concerns expressed by parents about certain inappropriate behaviours of children towards animals, impacting animal welfare, as reported in studies by Burgoyne et al. (2014), Carlisle (2014) and Harwood et al. (2019).

Based on the results obtained, it can be concluded that AAIs are perceived by parents of children with neurodevelopmental disorders as beneficial when considering their effects on different areas of development. These benefits also extend to the family environment, as positive changes in children and adolescents have an impact on parents’ well-being.

## Data Availability

Not applicable.
